# Molecular Determinants of the Kinetic Binding Properties of Antihistamines at the Histamine H_1_ Receptors

**DOI:** 10.3390/ijms22052400

**Published:** 2021-02-27

**Authors:** Hayato Akimoto, Yoshihiro Uesawa, Shigeru Hishinuma

**Affiliations:** 1 Department of Pharmacodynamics, Meiji Pharmaceutical University, 2-522-1 Noshio, Kiyose, Tokyo 204-8588, Japan; d206901@std.my-pharm.ac.jp; 2 Department of Medical Molecular Informatics, Meiji Pharmaceutical University, 2-522-1 Noshio, Kiyose, Tokyo 204-8588, Japan; uesawa@my-pharm.ac.jp

**Keywords:** antihistamine, affinity, association rate constant, dissociation rate constant, enthalpy, entropy, H_1_ receptor

## Abstract

The binding affinity of ligands for their receptors is determined by their kinetic and thermodynamic binding properties. Kinetic analyses of the rate constants of association and dissociation (*k*_on_ and *k*_off_, respectively) of antihistamines have suggested that second-generation antihistamines have a long duration of action owing to the long residence time (1/*k*_off_) at the H_1_ receptors. In this study, we examined the relationship between the kinetic and thermodynamic binding properties of antihistamines, followed by an evaluation of the structural determinants responsible for their kinetic binding properties using quantitative structure–activity relationship (QSAR) analyses. We found that whereas the binding enthalpy and entropy might contribute to the increase and decrease, respectively, in the *k*_off_ values, there was no significant relationship with the *k*_on_ values. QSAR analyses indicated that *k*_on_ and *k*_off_ values could be determined by the descriptors FASA_H (water-accessible surface area of all hydrophobic atoms divided by total water-accessible surface area) and vsurf_CW2 (a 3D molecular field descriptor weighted by capacity factor 2, the ratio of the hydrophilic surface to the total molecular surface), respectively. These findings provide further insight into the mechanisms by which the kinetic binding properties of antihistamines are regulated by their thermodynamic binding forces and physicochemical properties.

## 1. Introduction

Antihistamines, antagonists/inverse agonists of histamine H_1_ receptors, are widely used for the treatment of allergies such as allergic rhinitis and allergic dermatitis [1,2]. Antihistamines are usually divided into two generations, first and second, with most second-generation antihistamines having fewer side effects such as sedation and hypnosis owing to less penetration into the brain [3,4,5,6]. The binding affinity (the dissociation constant, *K*_d_) of antihistamines for H_1_ receptors is known to be determined by their kinetic binding parameters (the rate constants of association and dissociation, *k_o_*_n_ and *k*_off_, respectively) based on the equation, *K*_d_ = *k*_off_/*k*_on_. It has been recently revealed that second-generation antihistamines show a long duration of action owing to a long residence time (1/*k*_off_,) at the H_1_ receptors [7,8,9,10,11,12,13,14]. Thus, the kinetic binding parameters of antihistamines are important for determining their efficacy in vivo.

On the other hand, the binding affinity of antihistamines is also known to be thermodynamically determined by their binding enthalpy (∆*H*°) and entropy (−*T*∆*S*°) based on the equation, ∆*G*° = ∆*H*° − *T*∆*S*° = *RT*ln*K*_d_ [15,16,17,18,19,20]. Second-generation antihistamines have been determined to bind to H_1_ receptors via a stronger binding entropy than first-generation antihistamines [18]. However, the relationship between the kinetic and thermodynamic binding properties of antihistamines is unclear. Therefore, it is of interest to examine how the long residence time of antihistamines could be determined by their thermodynamic binding properties. In this study, we evaluated the relationship between previously reported kinetic binding parameters (*k*_on_ and *k*_off_) of antihistamines [7,8,9,10,11,12] and their thermodynamic binding parameters (∆*H*° and −*T*∆*S*°) obtained in our laboratory [18]. Furthermore, we evaluated the structural determinants responsible for the kinetic binding properties of antihistamines through quantitative structure–activity relationship (QSAR) analyses.

## 2. Results and Discussion

### 2.1. Relationship between the Kinetic and Thermodynamic Binding Parameters of Antihistamines

**Figure 1:**
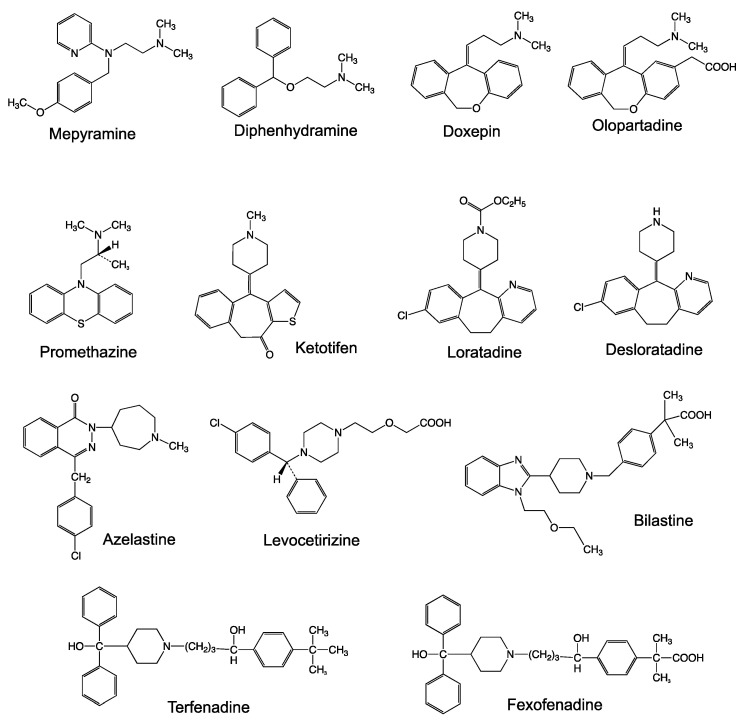
Chemical structure of the antihistamines evaluated in this study.

**Figure 2:**
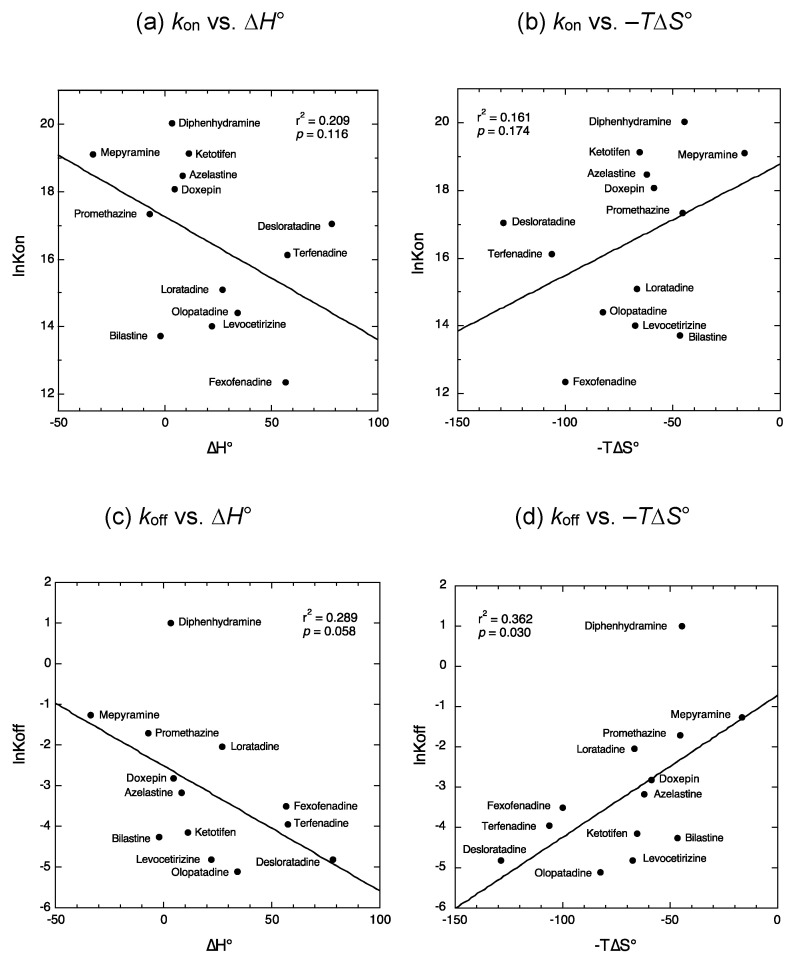
Relationship between the kinetic and thermodynamic binding parameters of antihistamines. Relationship between the values of ln*k*_on_ vs. ∆*H*°(**a**), ln*k*_on_ vs. −*T*∆*S*° (**b**), ln*k*_off_ vs. ∆*H*° (**c**), and ln*k*_off_ vs. −*T*∆*S*° (**d**). A significant relationship was observed between the values of ln*k*_off_ vs. −*T*∆*S*° (**d**). *k*_on_: rate constant of association; *k*_off_: rate constant of dissociation.

We first examined the relationship between the previously determined kinetic binding parameters (*k*_on_ and *k*_off_) of antihistamines [7,8,9,10,11,12] (Figure 1 and Table 1) and the thermodynamic binding parameters (∆*H*° and −*T*∆*S*°) obtained in our laboratory [18]. There was no significant relationship between the values of *k*_on_ and the thermodynamic binding parameters (Figure 2a,b). In contrast, the *k*_off_ value appeared to decrease concomitantly with decreases in the values of −*T*∆*S*°, i.e., increases in the entropy-dependent binding forces (Figure 2d: *p* = 0.030). Conversely, the *k*_off_ values tended to increase concomitantly with decreases in the values of ∆*H*°, i.e., increases in the enthalpy-dependent binding forces, although not significantly (Figure 2c: *p* = 0.058). These results suggested that the kinetic and thermodynamic binding parameters might differentially determine the binding affinity for antihistamines, although the k_off_ values appeared to be related in part to the thermodynamic binding properties.

### 2.2. QSAR Analyses to Estimate the Structural Determinants of the Kinetic Binding Properties of Antihistamines

**Figure 3:**
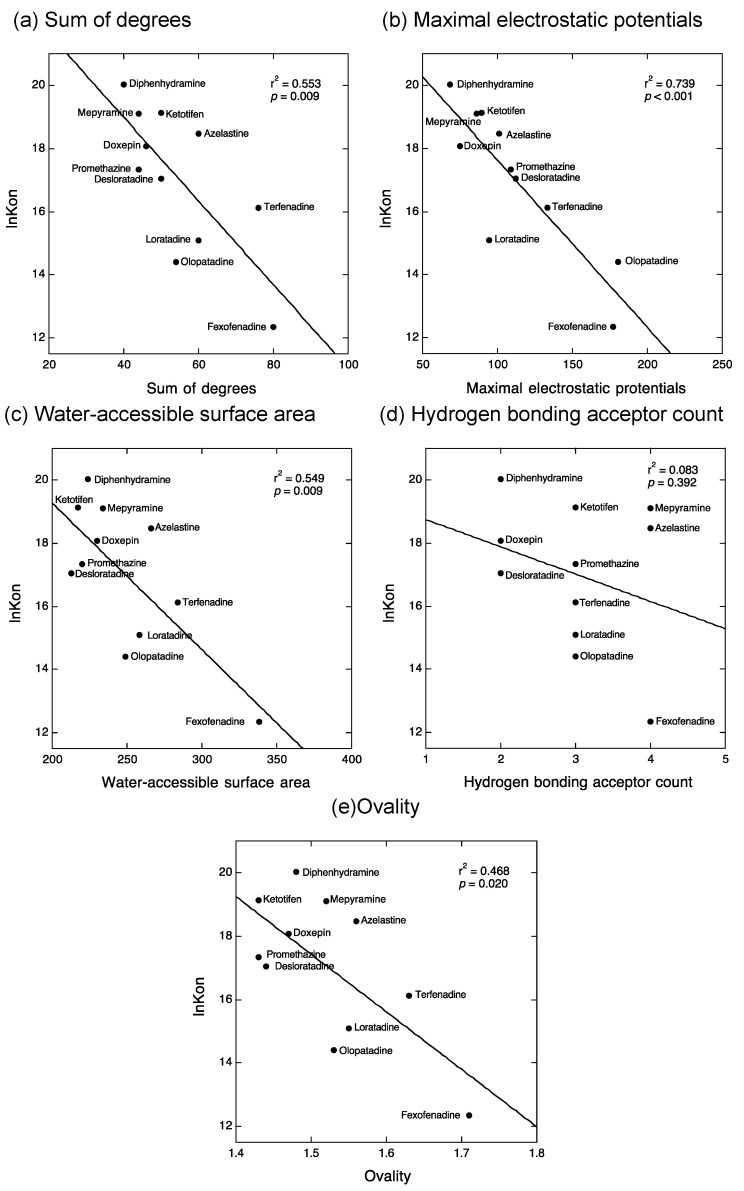
Relationship between the *k*_on_ values of antihistamines and their physicochemical properties involved in thermodynamic binding parameters. Relationship of the values of ln*k*_on_ with sum of degree (**a**), maximal electrostatic potentials (**b**), water-accessible surface area (**c**), hydrogen bonding acceptor count (**d**), and ovality (**e**). A significant relationship was observed for all parameters except between the values of ln*k*_on_ and the hydrogen bonding acceptor count (**d**). *k*_on_: rate constant of association.

**Figure 4:**
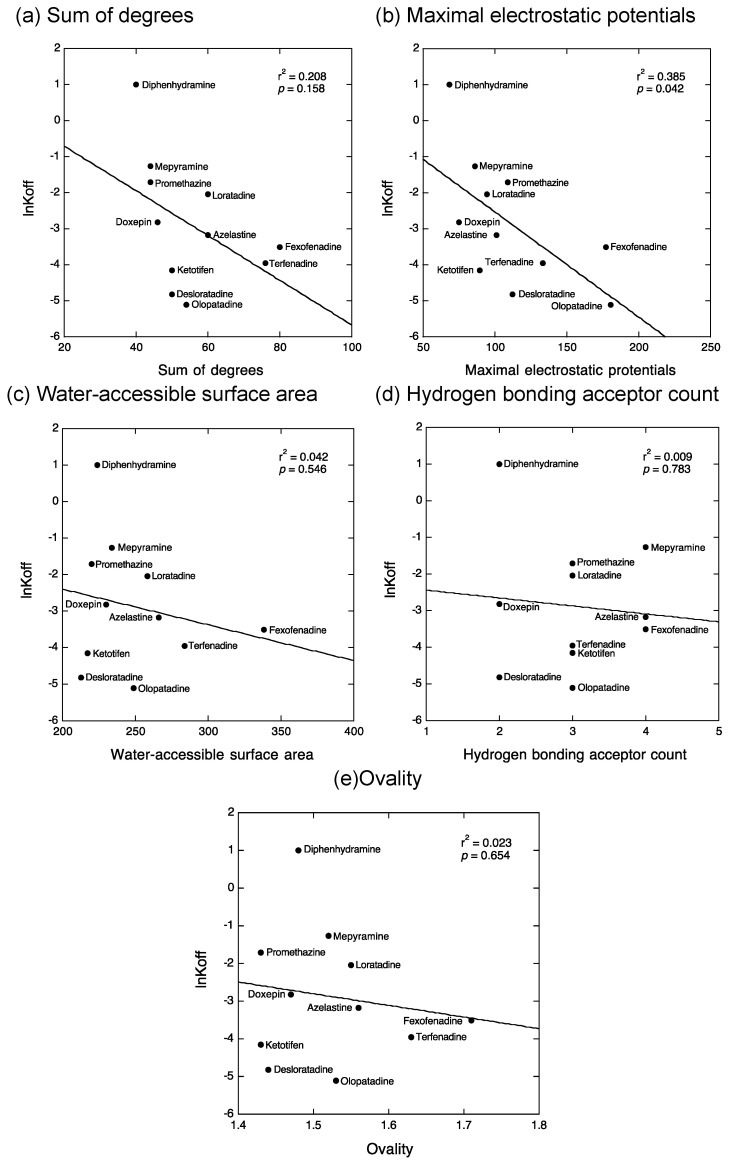
Relationship between the *k*_off_ values of antihistamines and their physicochemical properties involved in thermodynamic binding parameters. Relationship of the values of ln*k*_off_ with sum of degree (**a**), maximal electrostatic potentials (**b**), water-accessible surface area (**c**), hydrogen bonding acceptor count (**d**), and ovality (**e**). A significant relationship was observed only between the values of ln*k*_off_ and maximal electrostatic potentials (**b**). *k*_off_: rate constant of dissociation.

Our previous QSAR analyses indicated that the binding enthalpy and entropy of antihistamines are determined by five physicochemical properties of antihistamines, i.e., the sum of degrees (the sum of the number of non-hydrogen atoms bound to the compounds), maximal electrostatic potentials, water-accessible surface area, hydrogen binding acceptor count, and ovality (the surface area of a sphere equal to the solvent-excluded volume of the molecule) [18]. We first checked whether these five physicochemical properties of antihistamines might be involved in determining their kinetic binding parameters. Interestingly, four of the five parameters, except for the hydrogen bonding acceptor count, were significantly related to the *k*_on_ values (Figure 3) despite the lack of a significant relationship between *k*_on_ and thermodynamic binding parameters (Figure 2a,b). In contrast, only the maximal electrostatic potentials of antihistamines were significantly related to the *k*_off_ values (Figure 4). Thus, it was revealed that increases in the values of the sum of degrees, water-accessible surface area, and ovality of antihistamines were related to decreases in their *k*_on_ values and that increases in the values of maximal electrostatic potentials of antihistamines were related to decreases in their *k*_on_ and *k*_off_ values.

As these physicochemical properties of antihistamines were identified as the structural determinants responsible for the thermodynamic binding properties, we subsequently performed QSAR analyses designed specifically to explore the structural determinants responsible for the kinetic binding properties of antihistamines.

**Figure 5:**
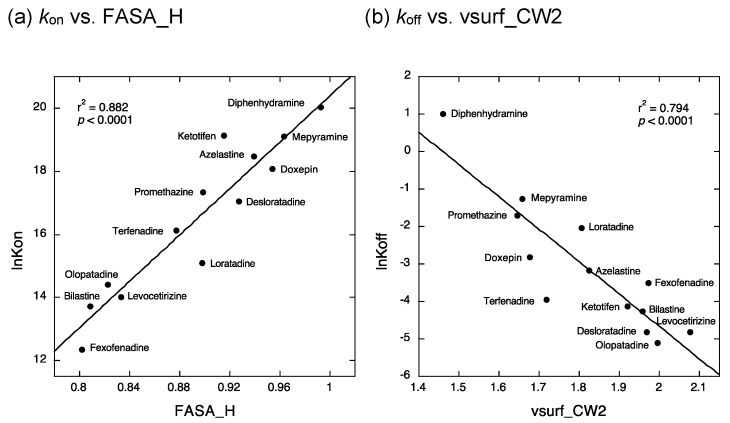
Quantitative structure–activity relationship (QSAR) analyses to identify the physicochemical properties of antihistamines that determine their kinetic binding parameters. Two descriptors, FASA_H (water-accessible surface area of all hydrophobic atoms divided by total water-accessible surface area) and vsurf_CW2 (a 3D molecular field descriptor weighted by capacity factor 2, the ratio of the hydrophilic surface to the total molecular surface) were identified as the structural determinants responsible for the values of *k*_on_ (**a**) and *k*_off_ (**b**), respectively. *k*_on_: rate constant of association; *k*_off_: rate constant of dissociation.

We found that the *k*_on_ values of antihistamines could be determined by their FASA_H values (water-accessible surface area of all hydrophobic atoms divided by total water-accessible surface area) with the equation, ln*k*_on_ = (36.76248 × FASA_H) − 16.3694 (r^2^ = 0.882, *p* < 0.0001) (Figure 5a). Thus, the *k*_on_ values of antihistamine appeared to increase concomitantly with increases in FASA_H. As FASA_H is the ratio of the molecular surface area of hydrophobic atoms, ligand molecules with large values of this descriptor have highly hydrophobic properties. That is, the *k*_on_ values of antihistamines might be well predicted by this descriptor, which correlates with the hydrophobicity of ligand molecules.

In addition, we found that the *k*_off_ values of antihistamines could be determined by the vsurf_CW2 values (a 3D molecular field descriptor weighted by capacity factor 2, the ratio of the hydrophilic surface to the total molecular surface) with the equation, ln*k*_off_ = (−8.6390831 × vsurf_CW2) + 12.612713 (r^2^ = 0.794, *p* < 0.0001) (Figure 5b). Thus, the *k*_off_ values of antihistamines appeared to decrease concomitantly with increases in the values of vsurf_CW2. vsurf_CW2 is a descriptor that convolves the three-dimensional shape of ligand molecules and the ratio of hydrophilic functional groups such as OH and NH groups to the molecular surface. That is, the *k*_off_ values of antihistamines might be well predicted by this descriptor, which represents the fitting of the hydrophilic molecular entity in the ligand-binding pocket of the receptor. It should be noted that the *k*_on_ and *k*_off_ values of antihistamines could be differentially predicted by these two descriptors.

In conclusion, this study revealed the relationship between the kinetic and thermodynamic binding properties of antihistamines, which may provide further insight into the mechanisms by which the affinities of ligands for their receptors are regulated by their kinetic and thermodynamic binding forces. Furthermore, the study revealed the structural determinants responsible for the kinetic binding properties of antihistamines, which may also provide useful information on the concept of ideal antihistamines from the viewpoint of the immediate and sustained effects of antihistamines.

## 3. Materials and Methods

### 3.1. Relationship between the Kinetic and Thermodynamic Binding Parameters of Antihistamines

The relationship between previously reported kinetic binding parameters (*k*_on_ and *k*_off_) of antihistamines [7,8,9,10,11,12] and their thermodynamic binding parameters (∆*H*° and −*T*∆*S*°) obtained in our laboratory [18] was evaluated by a simple liner regression model using KaleidaGraph (Synergy Software, Reading, PA, USA) (Figure 2). The relationship between the kinetic binding parameters of antihistamines and their five physicochemical properties (i.e., the sum of degrees, maximal electrostatic potentials, water-accessible surface area, hydrogen binding acceptor count, and ovality), which were identified as determinants for the thermodynamic binding properties of antihistamines in our previous study [18], was further evaluated by a simple liner regression model using KaleidaGraph (Figure 3 and Figure 4). *p* < 0.05 was considered significant.

### 3.2. QSAR Analyses to Estimate the Structural Determinants of the Kinetic Binding Properties of Antihistamines

The 3D-structure of each chemical structure was drawn using Marvin Sketch 18.10.0 (ChemAxon, Budapest, Hungary, http://www.chemaxon.com (accessed on 1 February 2021)), and optimized using Toxicity Predictor [21]. Energy minimization calculations were performed using the Merck Molecular Force Field. Molecular descriptors were calculated using Molecular Operating Environment version 2019.0101 (Chemical Computing Group Inc., Quebec, QC, Canada). Statistical analysis was performed using JMP Pro version 15.0.0 software (SAS Institute Inc., Cary, NC, USA). Applying the QSAR concept established by Hansch and Fujita [22], we conducted Pearson’s correlation analyses using the natural logarithms of *k*_on_ and *k*_off_ values and molecular descriptors as objective and explanatory variables, respectively. Based on the coefficient of determination (r^2^) and significance probability (*p*) calculated from the correlation analyses, descriptors that best explained the objective variable were selected from 143 kinds of descriptors included in the Molecular Operating Environment (supplementary materials). Descriptors with collinearity, r^2^ between the descriptors, greater than 0.95 and with standardized entropies of less than 0.3 were excluded from the 344 descriptors. As a result, 143 descriptors were selected, and used for the QSAR analyses. In addition, regression diagnostics on the scatter plot of the selected descriptors and the target variable were performed (Figure 5). By evaluating the normal distribution of the residuals between the calculated and experimental values, the results of the regression diagnostics were confirmed to be normal. The structural and physicochemical meanings of the descriptors were obtained by referring to the online manual of the Molecular Operating Environment.

## Figures and Tables

**Table 1:** Kinetic binding parameters for antihistamines used in this study.

Antihistamines	*k*_on_ (10^6^ min^−1^·M^−1^)	*k*_off_ (min^−1^)	References ^1^
Loratadine	3.6	0.13	Gillard et al. [7]
Terfenadine	10	0.019	Gillard et al. [7]
Azelastine	106	0.042	Slack et al. [8]
Ketotifen	204	0.016	Kanamitsu et al. [9]
Desloratadine	25	0.008	Bosma et al. [10]
Doxepin	70	0.06	Bosma et al. [10]
Levocetirizine	1.2	0.008	Bosma et al. [10]
Mepyramine	200	0.28	Bosma et al. [10]
Olopatadine	1.8	0.006	Bosma et al. [10]
Bilastine	0.9	0.014	Bosma et al. [11]
Diphenhydramine	500	2.70	Bosma et al. [11]
Fexofenadine	0.23	0.03	Bosma et al. [11]
Promethazine	33.7	0.18	Stoddart et al. [12]

^1^ Values of *k*_on_ and *k*_off_ for antihistamines were taken from these references.

## Data Availability

Not applicable.

## Supplementary Materials

The following are available online at https://www.mdpi.com/1422-0067/22/5/2400/s1.
